# Supplementary material for: Metric clusters in evolutionary games on scale-free networks
Source: Nat Commun. 2017 Dec 1;8:1888. doi: 10.1038/s41467-017-02078-y (PMC5709505; doi:10.1038/s41467-017-02078-y)
Supplement: Supplementary file 1 — Supplementary Information [file 41467_2017_2078_MOESM1_ESM.pdf]

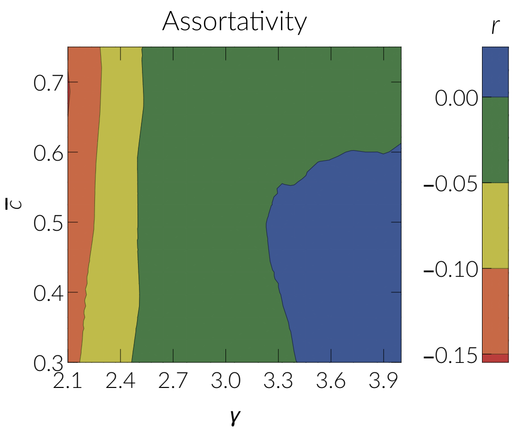

Supplementary Figure 1. **Assortativity of the considered networks.** Assortativity coefficient r as defined in [1] for the synthetic networks as generated in Fig. 4 in the manuscript.

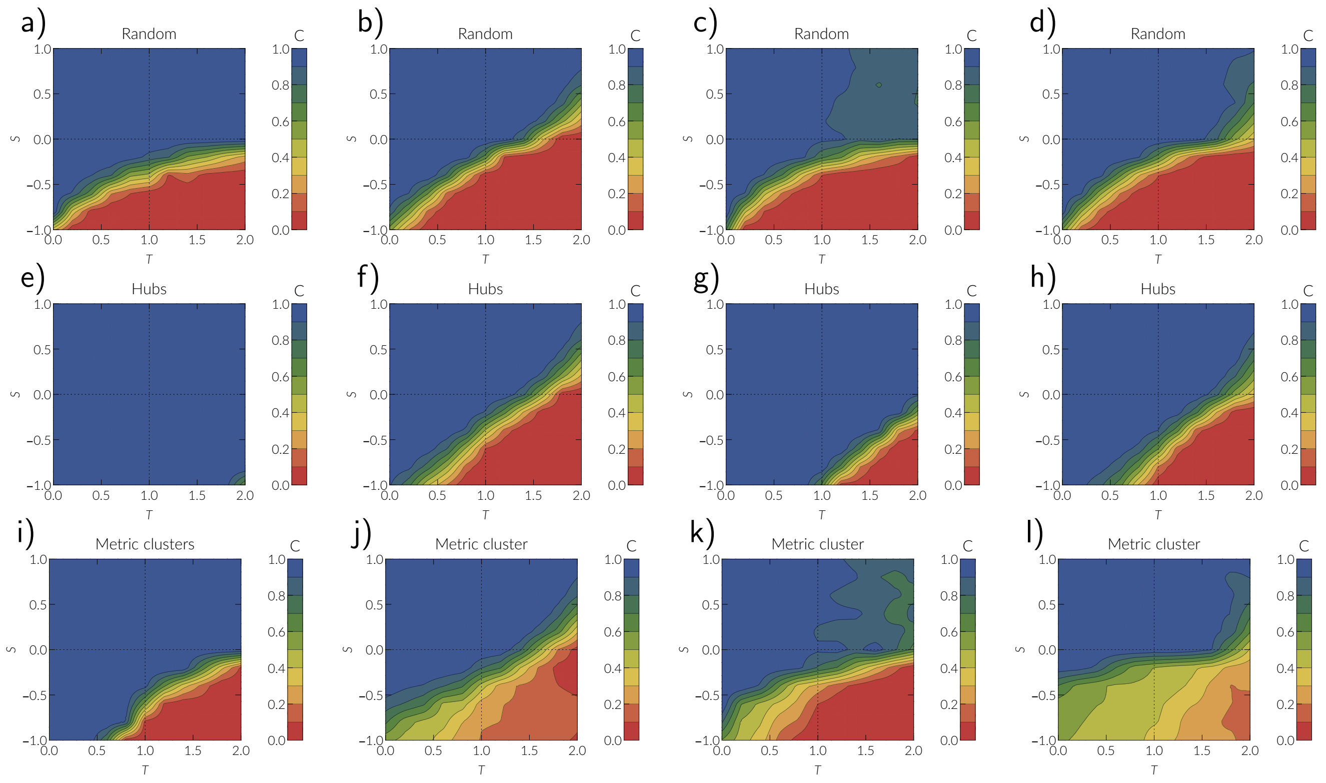

Supplementary Figure 2. **Final cooperation (color coded) averaged over** 50 **realizations of the system. a-d)** Random initial assignment of cooperators. **a)** For the Internet IPv6 network. **b)** For the arXiv collaboration network. **c)** Synthetic network with $N = 2 \cdot 10^4$ nodes, power-law exponent $\gamma = 2.4$, mean degree $\langle k \rangle \approx 6$, and clustering $\bar{c} = 0.5$. **d)** The same as before but for networks with power-law exponent $\gamma = 2.9$ and clustering $\bar{c} = 0.6$. **e-f)** The same as (a-d) but for cooperators initially assigned to the hubs. **i-l)** The same as (a-d) but for initial cooperators allocated into a metric cluster.

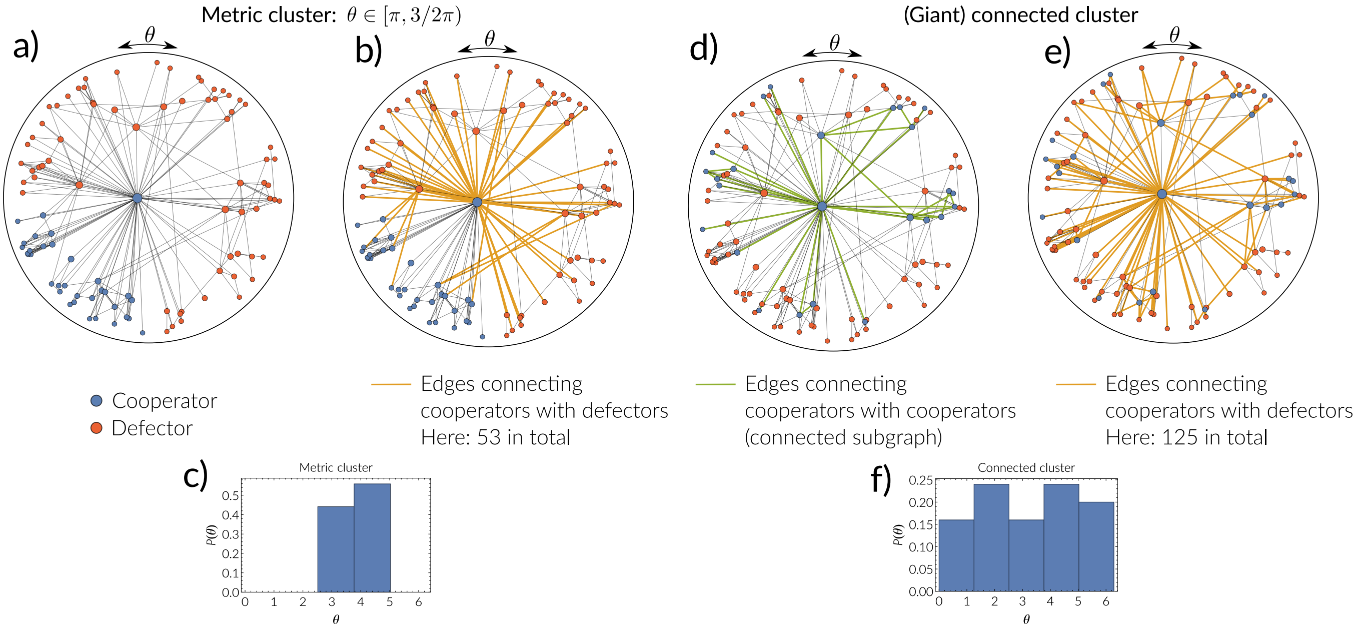

Supplementary Figure 3. **Connected vs. metric clusters.** Illustration of the difference between a connected cluster and a metric cluster using an explicit example network generated with the model described in the manuscript with $N = 100$ nodes.. **a)** A metric cluster comprising nodes with a similarity (angular) coordinate $\theta \in [\pi, 3/2\pi)$ are marked by blue. **b)** The same as in a) but now all links that connect a defector (blue) with a cooperator (red) are marked by yellow. In total, there are 53 such links in the example. **c)** Distribution of similarity coordinates θ among the nodes within the metric cluster. **d)** Starting form the same network as in (A), 25 nodes are assigned into a connected cluster using the procedure described in the manuscript. The links connecting cooperators with other cooperators are marked by green. Note that these links connect all of the blue nodes, hence the blue nodes form a connected component (or connected cluster, network cluster, giant cluster). **e)** The same as d) but now links that connect cooperators with defectors are marked by yellow. These links are significantly more abundant as compared to the case of metric clusters, with a total of 125 such links (the whole network contains 244 links). **f)** Histogram of the distribution of similarity (angular) coordinates θ of the blue nodes in f) and e). The similarity coordinates are broadly distributed (for a large network, this distribution would become uniform).

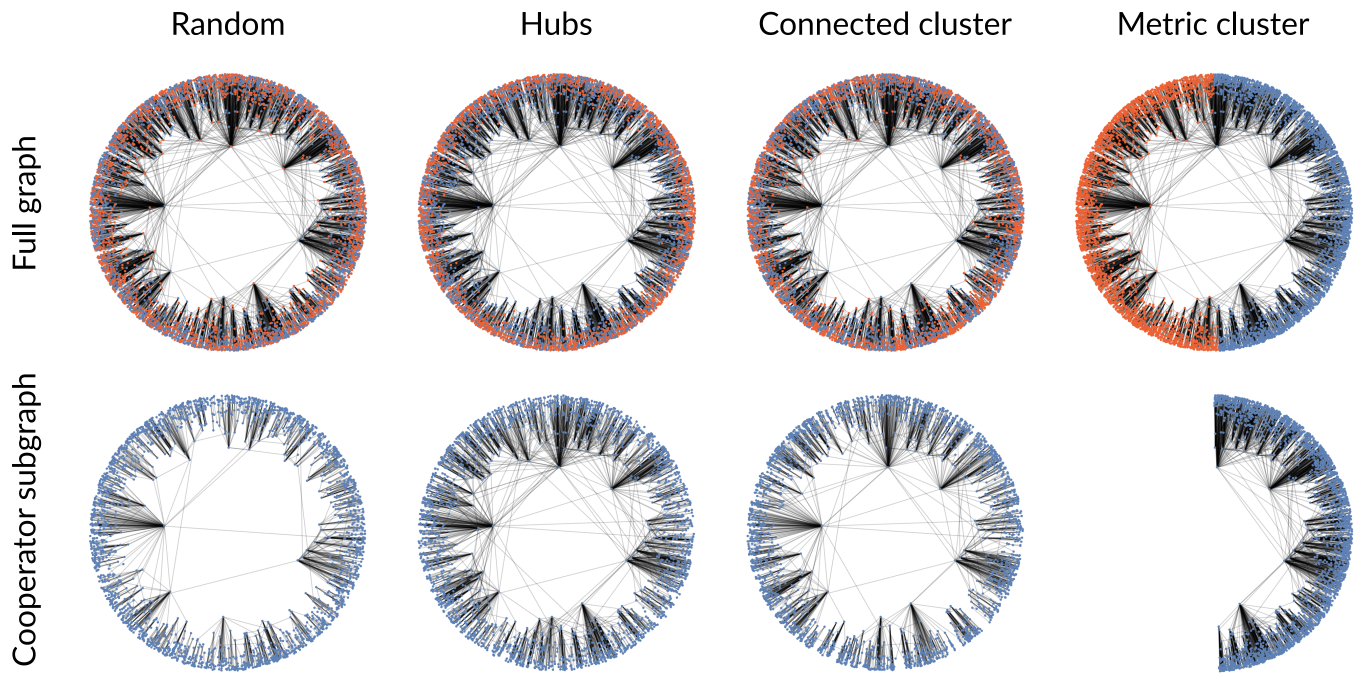

Supplementary Figure 4. **Initial assignment.** Examples of the initial assignment of cooperators as described in the text. Top row shows the whole network, were blue denotes cooperators and red defectors. Bottom row shows only the subgraph of cooperating nodes.

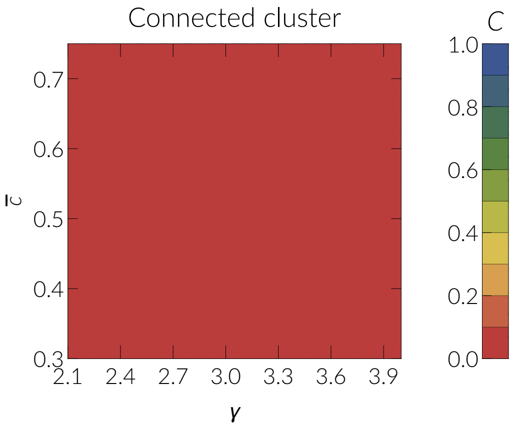

Supplementary Figure 5. **Connected cluster.** Final (after $2 \cdot 10^5$ update steps) density of cooperators (color coded) for the prisoner's dilemma ($T = 1.5$ and $S = -0.5$) averaged over 50 realizations as a function of the degree distribution power-law exponent γ and mean local clustering $\bar{c}$. Networks have $N = 2 \cdot 10^4$ nodes and a mean degree $\langle k \rangle \approx 6$. The initial density of cooperators is always $c(0) = 0.5$. Initial cooperators were assigned into a connected cluster.

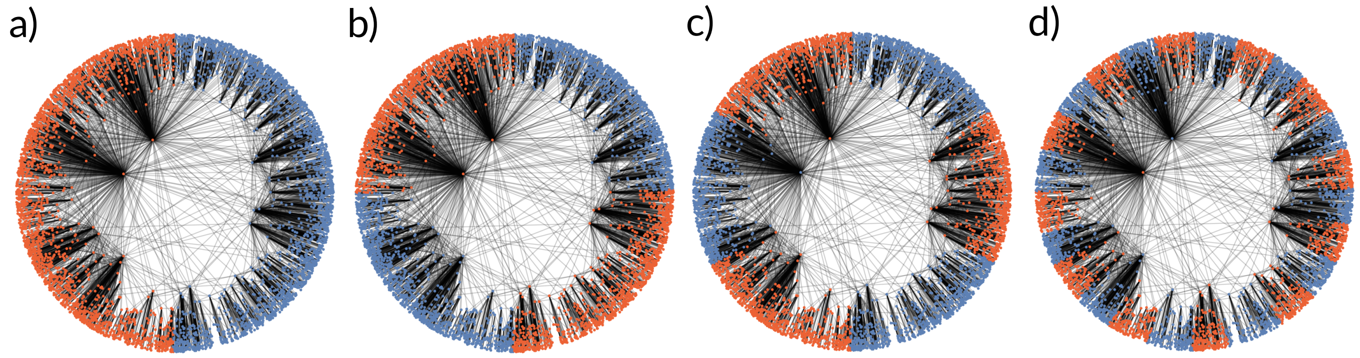

Supplementary Figure 6. **Assignment into multiple metric clusters. a)** One cluster. **b)** Two clusters. **c)** Three clusters. **d)** 12 clusters.

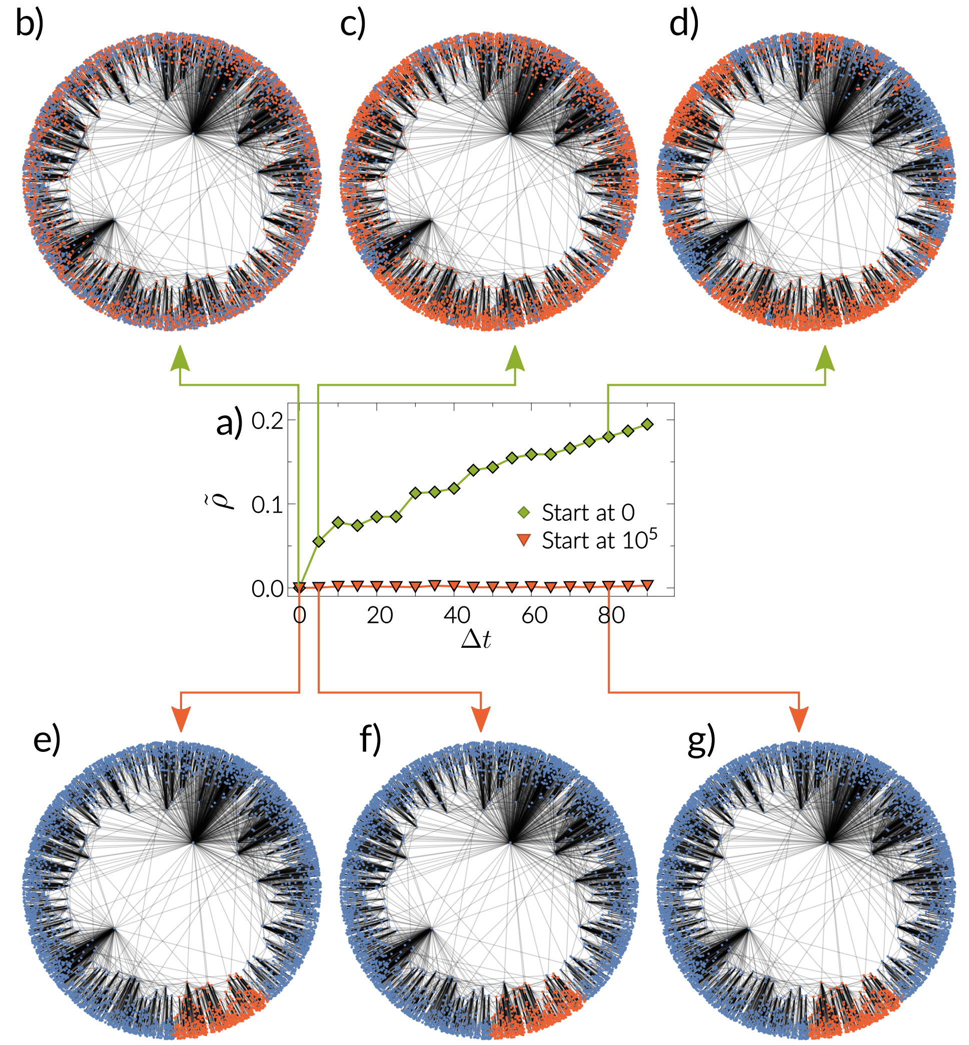

Supplementary Figure 7. **Stationary state. a)** KS-statistics $\tilde{\rho}$ as defined in Eq. (1) as a function of the difference of update steps starting at $t = 0$ (green) and $t = 10^5$ (red). Results are for a single realization for $T = 1.2$ and $S = -0.2$, a synthetic network with $N = 5000$ nodes, power law exponent $\gamma = 2.8$, mean local clustering $\bar{c} \approx 0.5$, and randomly assigned initial cooperators. **b-d)** State of the system at times $t = (0, 5, 80)$ respectively. Blue nodes are cooperators, and red ones denote defectors. **e-g)** State of the system at times $t = (10^5, 10^5 + 5, 10^5 + 80)$ respectively.

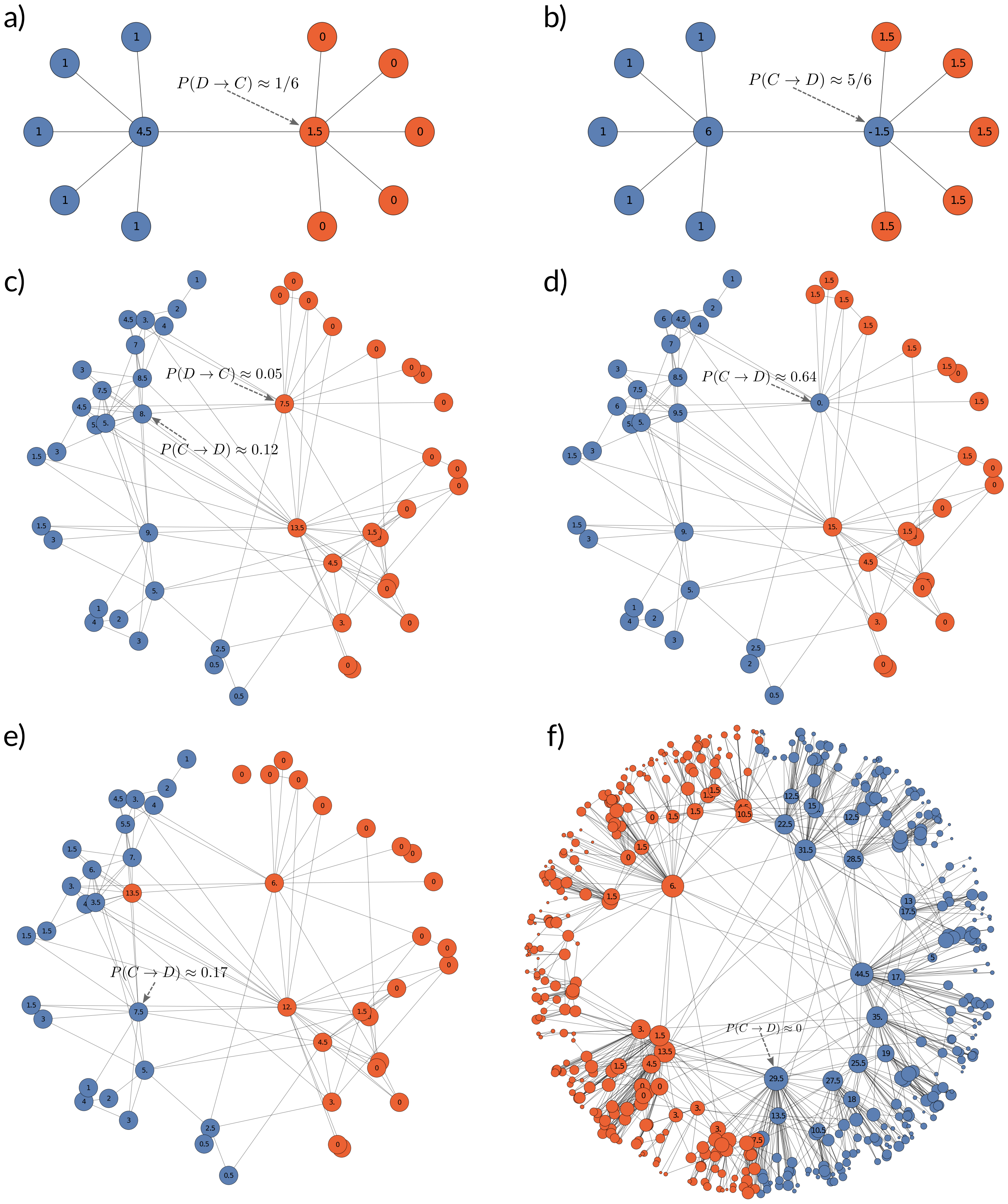

Supplementary Figure 8. **Toy examples.** Simple toy example for the prisoner's dilemma game ($T = 1.5$ and $S = -0.5$). Cooperators are marked by blue and defectors by red. Numbers denote the payoffs earned in the respective configuration. **a)** Double star graph which consist of two star graphs whose hubs are connected. The left star graph can be thought of as a proxy for a metric cluster of cooperators (blue nodes) and the right star graph are neighboring defectors (red nodes). **b)** The same graph after the right hub switches to cooperation. **c-e)** Illustrations similar to a and b but for a small network ($N = 50$) generated with the model described in the main text. **f)** An illustration similar to c but for a larger network with $N = 500$ nodes.

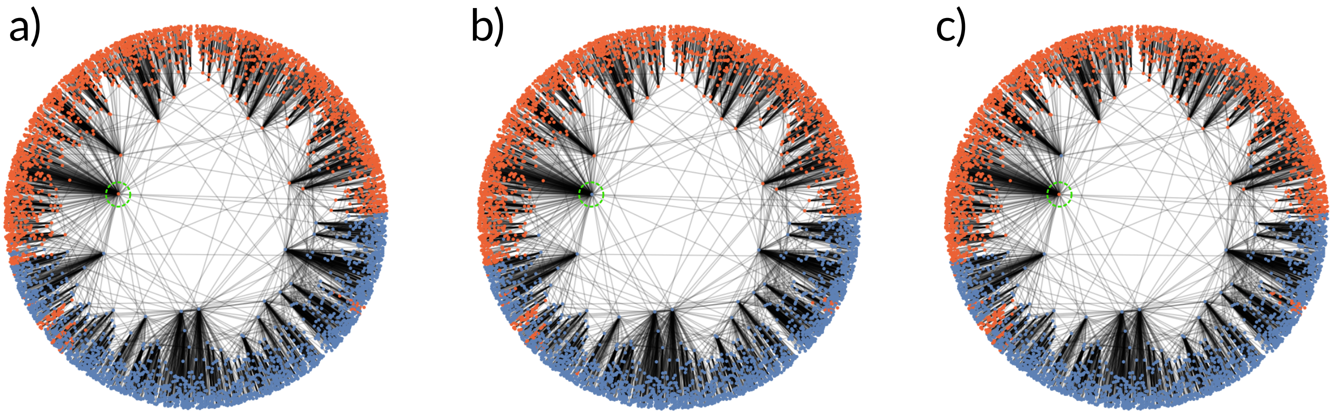

Supplementary Figure 9. **Simulation results.** Three snapshots from the evolution shown in Supplementary Video 1. **a)** $T = 2.3 \cdot 10^5$. **b)** $T = 2.4 \cdot 10^5$. **c)** $T = 2.5 \cdot 10^5$.

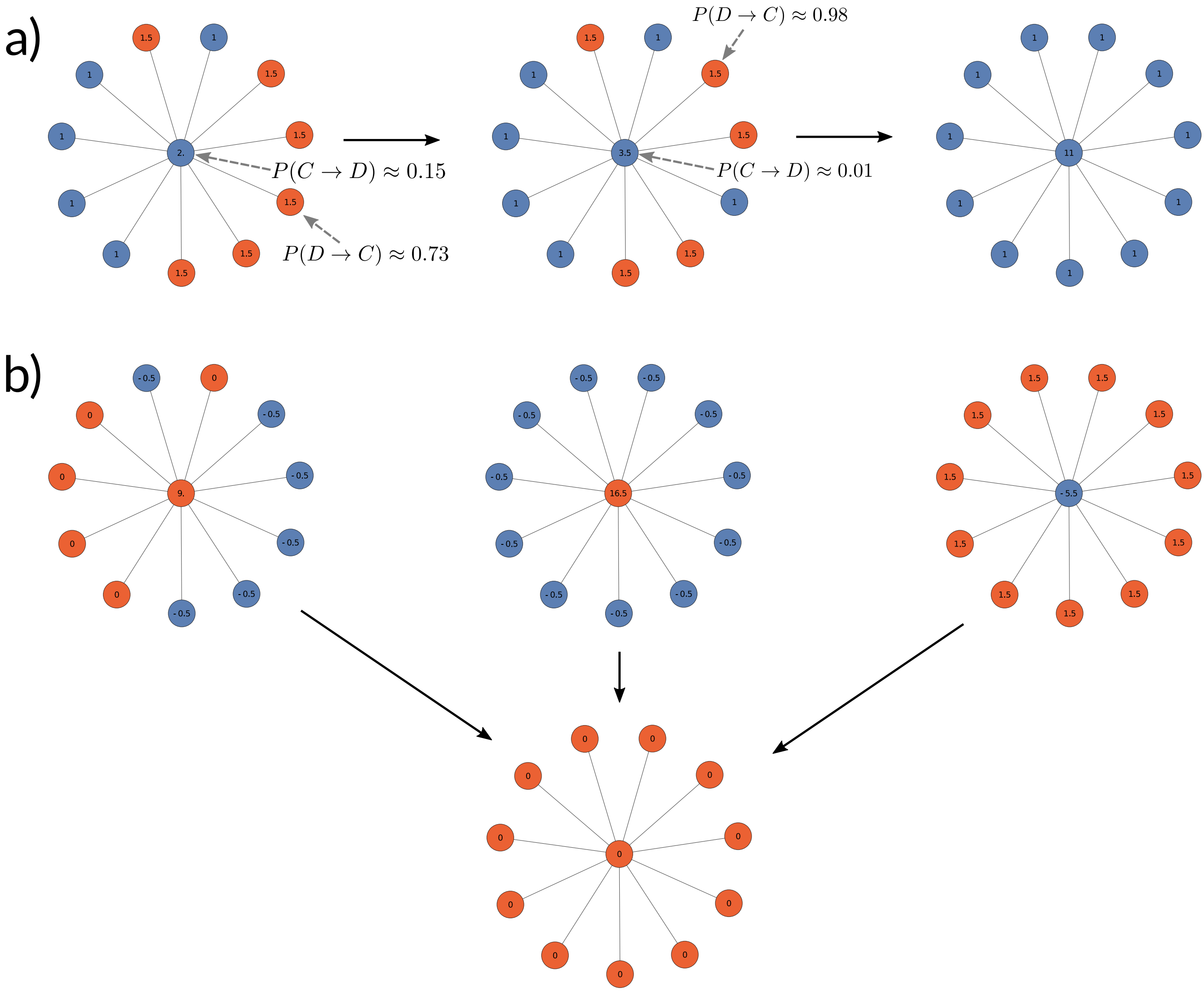

Supplementary Figure 10. **Star network.** Simple toy example for the prisoner's dilemma game ($T = 1.5$ and $S = -0.5$) using a star network. Cooperators are marked by blue and defectors by red. **a)** Cooperative hubs can drive the system towards cooperation. **b)** Examples where eventually defection is likely to prevail.

SUPPLEMENTARY NOTE 1
ASSORTATIVITY

We note that the empirical networks considered in the main manuscript show clearly different assortativity coefficients. The Internet TPv6 topology has $r = -0.29$ and hence is significantly disassortative whereas the Arxiv network is clearly assortative with $r = 0.24$. Nevertheless, for the values of γ and $\bar{c}$ where the interesting behavior takes place (Fig. 4 of the main manuscript), the synthethic networks exhibit a nearly constant assortativity coefficient close to zero, $r \in (-0.05, 0)$. In particular, in Supplementary Figure 1 we show the assortativity coefficient r as defined in [1] for the synthetic networks generated as a function of their properties, i.e. the power-law exponent γ and the mean local clustering coefficient $\bar{c}$. We conclude that assortativity cannot explain the observed behavior.

SUPPLEMENTARY NOTE 2
INITIAL CONDITIONS, CONNECTED CLUSTERS, AND METRIC CLUSTERS

Let us first explain the difference between a connected cluster and a metric cluster. A connected cluster, sometimes called giant cluster or giant connected component (GCC) is defined by the connectivity in the network [2]. A connected component is defined by a set of nodes $\mathcal{V}$ such that there exists a path in the network between any pair of nodes $i \in \mathcal{V}$, $j \in \mathcal{V}$ that only passes through nodes $l \in \mathcal{V}$. In other words, each pair of nodes in the cluster is connected by a path that only goes through nodes that form part of the cluster.

The metric clusters considered in the manuscript are sets of nodes whose similarity (angular) coordinates θ lie within a certain interval. The coordinate θ abstracts a similarity measure that cannot be observed directly by looking at the topology of the network, but only becomes visible by embedding the network into hidden metric spaces (see [3, 4]). As explained in the main manuscript, we use an embedding into hyperbolic space [5, 6], where each node has a popularity (radial) coordinate r and the aforementioned similarity (angular) coordinate θ. The angular distance, $\Delta\theta_{ij} = \pi - |\pi - |\theta_i - \theta_j||$, is then a measure of the similarity between nodes i and j. For example, a lawyer would probably be closer to a judge in this similarity measure as compared to a bus driver, even if the lawyer and the judge are not connected themselves. For a comprehensive overview and discussion of this similarity measure, we refer the reader to [5, 6].

Now, let us consider an explicit example to illustrate the difference between a connected and a metric cluster using a small network generated with the model described in the manuscript. Supplementary Figure 3a shows an example of a metric cluster which contains nodes with a similarity (angular) coordinate $\theta \in [\pi, 3/2\pi)$ (marked by blue). This condition is reflected by the distribution of similarity coordinates θ among the nodes within the metric cluster in Supplementary Figure 3c. Supplementary Figure 3d shows an example of a connected cluster, which contains the nodes marked by blue. Links connecting cooperators with cooperators (blue nodes with blue nodes) are marked by green. Each pair of cooperators is connected by a path using only the green edges, hence, the cooperators form a unique connected cluster. The distribution of the similarity coordinate shown in Supplementary Figure 3f is significantly different from the case of the metric cluster, and for a larger network this distribution will become uniform. An important difference between these two cases is the number of links that connect cooperators with defectors. In Supplementary Figure 3b we highlight these links by yellow for the example of the metric cluster. These intercluster links form mainly at the border of the cluster (although this effect would be more pronounced for a larger network). In this example, there are 53 intercluster links. However, in the case of the connected cluster, the intercluster links are less constraint and occur in the whole angular space. In the particular example considered here, there are 125 intercluster links, more than twice as much as in the case of the metric cluster.

Supplementary Figure 4 shows an explicit example of the initial assignment of cooperators performed by the procedure explained in the manuscript. Note that in the case of assignment into a connected cluster, the subgraph of cooperators (shown in the bottom row of Supplementary Figure 4) forms a unique connected component.

Supplementary Figure 5 shows results similar to Fig. 4a in the main manuscript for initially assigning cooperators into a connected (or unique network [7]) cluster. The assignment procedure is described in the main manuscript and repeated here for the convenience of the reader: We start from the initial graph and randomly remove nodes until the size of the giant connected component (GCC) reaches $N/2$. The nodes that are now in the GCC are then assigned as cooperators in the original graph, and the remaining $N/2$ nodes are assigned as defectors. This procedure ensures that the initial cooperators form a connected subgraph. Note that a network cluster in general is not a metric cluster.

SUPPLEMENTARY NOTE 3
MULTIPLE METRIC CLUSTERS

In Supplementary Figure 6 we show initial conditions of assigning cooperators into multiple metric clusters. The assignment procedure is explained in the main manuscript and repeated here for the convenience of the reader: We sort all nodes by their angular coordinate θ. We now fix a number of distinct clusters, n_c, and assign the first $N/(2n_c)$ nodes as cooperators, the second $N/(2n_c)$ nodes as defectors, the third $N/(2n_c)$ as cooperators and so on.

SUPPLEMENTARY NOTE 4
STATIONARY STATE

Here, we present the value of the KS-statistics between different states of the evolution of the system. To this end, we define $\tilde{\rho}$ similar to $\bar{\rho}$ in the main manuscript as follows:

$$\tilde{\rho}(t_2, t_1) = \frac{c(t_2) + c(t_1)}{2} \rho_C(t_1, t_2) + \frac{d(t_2) + d(t_1)}{2} \rho_D(t_1, t_2) \,, \tag{1}$$

where we have defined

$$\rho_C(t_1, t_2) = \max_{\theta \in \{0, 2\pi\}} |C(\theta, t_1) - C(\theta, t_2)| \tag{2}$$

and

$$\rho_D(t_1, t_2) = \max_{\theta \in \{0, 2\pi\}} |D(\theta, t_1) - D(\theta, t_2)| \,. \tag{3}$$

In Supplementary Figure 7 we show the value of $\tilde{\rho}(t, 0)$ (green) and $\tilde{\rho}(t + 10^5, 10^5)$ (red) from $t = 0$ to $t = 90$. We observe that $\tilde{\rho}(t, 0)$ increases with t, which means that the angular distribution becomes increasingly different from the initial state as time increases. The value of $\tilde{\rho}(t + 10^5, 10^5)$ remains constant close to 0 since the system has reached a stationary state. This behavior is confirmed by the explicit plots of the state of the system at different times in Supplementary Figure 7.

SUPPLEMENTARY NOTE 5
SIMPLE TOY EXAMPLES

In this section, we explain how cooperation can be sustained. Therefore, we start using a very simple example, then we discuss small networks generated by the model described in the main manuscript, we proceed discussing the importance of size in an example of a larger network generated with the same model, and finally we connect these insights to the evolution of a realization of the system as presented in the main manuscript and Supplementary Videos.

In Supplementary Figure 8a we consider a double star graph [8], where we assume that the left star represents a cluster of cooperators and the right one neighboring defectors. The right hub, which is a defector, chooses the left hub as an imitation candidate with a probability equal to its inverse degree. Since the payoff difference is large, the probability to imitate is nearly one. Hence, the probability that the right hub switches to cooperation is $p(D \to C) \approx 1/6$. Assume now that this transition has occurred, and the right hub is a cooperator (Supplementary Figure 8b). The probability that the right hub selects one of the defecting leaves as an imitation candidate is given by one minus its inverse degree. Since again the payoff difference is large, the probability to imitate the strategy is nearly one. Hence, the probability that the right hub switches back to defection is $p(C \to D) \approx 5/6$. The probability that a leave node imitates the hub is given by $p(\Delta\pi)$ (see Eq. 2 in the main manuscript), which is smaller than 1% in this example. Therefore, in this example, the state of the right hub may fluctuate, but most of the time it will be defecting, and the leaves remain defectors. Hence, the clustered state does not take over the defectors.

Let us now consider a small network generated by the model described in the main manuscript. In Supplementary Figure 8c we show a configuration with a metric cluster of cooperators. Similar to the previous case, defecting hubs can become cooperative with a small probability (5% for the node highlighted in the figure). Once this happens (Supplementary Figure 8d), the probability is high that they again switch to defection (65% for the node highlighted in the figure). This explains why the cooperating cluster cannot invade the defectors. On the other hand, in this example the defectors can invade the cooperating cluster. A cooperating hub can become a defector (with 12% probability for the highlighted cooperating hub in Supplementary Figure 8c). Once this happens, the hub obtains a

high payoff and defection is likely to spread inside the cooperating cluster (Supplementary Figure 8e). Importantly, if the size of the system increases, the difference in payoff between cooperating and defecting hubs increases in favor for the former. As a consequence, the probability that a cooperating hub switches to defection quickly goes to zero (see Eq. 2 in the main manuscript), and the invasion by defectors becomes basically impossible (see Supplementary Figure 8f). This explains why larger clusters are more likely to survive (cf. Fig. 4g in the main manuscript).

Indeed, in the simulations we observe the fluctuations of the state of defecting hubs mentioned earlier. Supplementary Figure 9 shows three different snapshots of the evolution of the system from Supplementary Video 1. Indeed, in the second snapshot the hub which is marked by the green circle cooperates although its leaves are defectors. Most of the time, however, this hub defects, which can be seen in the Supplementary Video.

Let us now consider the case of very heterogeneous networks. In the case of $\gamma = 2$, the resulting networks are star-like, and here we consider a toy example of a small star network. If we start with half of the nodes as cooperators including the hub, the system is very likely to end up in full cooperation. In particular, in the initial state (Supplementary Figure 10a left), the probability that the hub becomes a defector is $p(C \rightarrow D) = \frac{n_D}{k} p(\Delta\pi) \approx 0.15$ (n_D is the number of defecting neighbors, k the degree of the hub). The probability that a leave node becomes cooperative is $p \approx 0.73$. Importantly, if one of the leave nodes switches to cooperation, this further favors cooperation, as now the probability of the cooperating hub to become a defector is approximatively 1%, and a leave node will switch to cooperation with a probability of approximatively 98% (Supplementary Figure 10a center). This simple example explains why hubs can drive the system towards a state of high cooperation if the network is sufficiently heterogeneous.

In Supplementary Figure 10b we show some examples where, using the same line of reasoning as before, it is highly likely that all nodes will eventually defect. These examples are half of the nodes are initially cooperative but the hub defects (left), the hub defects but the leaves cooperate (center), and the leaves defect but the hub cooperates (right).

SUPPLEMENTARY REFERENCES

[1] Mark Newman, *Networks: An Introduction* (Oxford University Press, Inc., New York, NY, USA, 2010).
[2] Mark Newman, Albert-László Barabási, and Duncan J. Watts, *The Structure and Dynamics of Networks* (Princeton, NJ: Princeton University Press, 2006).
[3] Fragkiskos Papadopoulos, Constantinos Psomas, and Dmitri Krioukov, "Network mapping by replaying hyperbolic growth," IEEE/ACM Transactions on Networking **23**, 198–211 (2015).
[4] Fragkiskos Papadopoulos, Rodrigo Aldecoa, and Dmitri Krioukov, "Network geometry inference using common neighbors," Phys. Rev. E **92**, 022807 (2015).
[5] Fragkiskos Papadopoulos, Maksim Kitsak, M. Ángeles Serrano, Marián Boguñá, and Dmitri Krioukov, "Popularity versus similarity in growing networks," Nature **489**, 537–540 (2012).
[6] Marián Boguñá, Fragkiskos Papadopoulos, and Dmitri Krioukov, "Sustaining the Internet with hyperbolic mapping." Nature communications **1**, 62 (2010).
[7] J. Gómez-Gardeñes, M. Campillo, L. M. Floría, and Y. Moreno, "Dynamical organization of cooperation in complex topologies," Phys. Rev. Lett. **98**, 108103 (2007).
[8] Francisco C Santos, Marta D Santos, and Jorge M Pacheco, "Social diversity promotes the emergence of cooperation in public goods games," Nat. **454**, 213–216 (2008).
