# Supplementary material for: Metric clusters in evolutionary games on scale-free networks
Source: Nat Commun. 2017 Dec 1;8:1888. doi: 10.1038/s41467-017-02078-y (PMC5709505; doi:10.1038/s41467-017-02078-y)
Supplement: Supplementary file 3 — Description of Additional Supplementary Files [file 41467_2017_2078_MOESM3_ESM.pdf]

Description of Additional Supplementary Files

File Name: Supplementary Movie 1
Description: Cluster formation

File Name: Supplementary Movie 2
Description: Hubs control

File Name: Supplementary Movie 3
Description: Hubs fail

File Name: Supplementary Movie 4
Description: Metric cluster sustained

File Name: Supplementary Movie 5
Description: Metric cluster fails
